# Safely reducing haemodialysis frequency during the COVID-19 pandemic

**DOI:** 10.1186/s12882-020-02172-2

**Published:** 2020-12-07

**Authors:** Michelle Da Silva Lodge, Thilini Abeygunaratne, Helen Alderson, Ibrahim Ali, Nina Brown, Constantina Chrysochou, Rosie Donne, Ibi Erekosima, Philip Evans, Emma Flanagan, Simon Gray, Darren Green, Janet Hegarty, Audrey Hyde, Philip A. Kalra, Elizabeth Lamerton, David Lewis, Rachel Middleton, David New, Robert Nipah, Donal O’Donoghue, Edmond O’Riordan, Dimitrios Poulikakos, Francesco Rainone, Maharajan Raman, James Ritchie, Smeeta Sinha, Grahame Wood, J. Tollitt

**Affiliations:** Department of Renal Medicine, Salford Royal NHS Trust, Stott Lane, Salford, M68HD UK

**Keywords:** COVID-19, Haemodialysis, Coronavirus, Mortality, Twice weekly, SARS-CoV-2

## Abstract

**Background:**

Patients undergoing haemodialysis (HD) are at higher risk of developing worse outcomes if they contract COVID-19. In our renal service we reduced HD frequency from thrice to twice-weekly in selected patients with the primary aim of reducing COVID 19 exposure and transmission between HD patients.

**Methods:**

Dialysis unit nephrologists identified 166 suitable patients (38.4% of our HD population) to temporarily convert to twice-weekly haemodialysis immediately prior to the peak of the COVID-19 pandemic in our area. Changes in pre-dialysis weight, systolic blood pressure (SBP) and biochemistry were recorded weekly throughout the 4-week project. Hyperkalaemic patients (serum potassium > 6.0 mmol/L) were treated with a potassium binder, sodium bicarbonate and received responsive dietary advice.

**Results:**

There were 12 deaths (5 due to COVID-19) in the HD population, 6 of which were in the twice weekly HD group; no deaths were definitively associated with change of dialysis protocol. A further 19 patients were either hospitalised and/or developed COVID-19 and thus transferred back to thrice weekly dialysis as per protocol. 113 (68.1%) were still receiving twice-weekly HD by the end of the 4-week project. Indications for transfer back to thrice weekly were; fluid overload (19), persistent hyperkalaemia (4), patient request (4) and compliance (1). There were statistically significant increases in SBP and pre-dialysis potassium during the project.

**Conclusions:**

Short term conversion of a large but selected HD population to twice-weekly dialysis sessions was possible and safe. This approach could help mitigate COVID-19 transmission amongst dialysis patients in centres with similar organisational pressures.

## Background

Severe Acute Respiratory Syndrome Coronavirus-2 has developed into a worldwide pandemic, with over 44 million documented cases and 1.1 million deaths worldwide. Medical comorbidities such as hypertension, diabetes mellitus, asthma, obesity and chronic kidney disease are reported as significant predictors of morbidity and mortality in COVID-19 patients [1, 2].

The necessary frequency of haemodialysis (HD) is particularly pertinent at a time of a worldwide pandemic [3]. There is no randomised study demonstrating a beneficial effect of thrice weekly dialysis over twice weekly dialysis. HD patients are an “at-risk” group who have worse outcomes if they contract COVID-19 [4–6]. UK Renal Registry data up to 7th October 2020 has reported 21.1% mortality for in centre HD patients who suffer COVID-19 [7]. Reducing frequency of dialysis for some patients who dialyse in-centre may minimise patient exposure to COVID-19, allow extra space between patients undergoing dialysis and help manage unprecedented HD staff sickness [8]. The counterpoint argument follows that dialysis reduction in patients with multimorbidity may increase overall morbidity, cardiovascular events and death, especially because of the longer interdialytic gap. This gap is notorious for being associated with higher risk of death and hospitalisation even in thrice weekly patients [9–11].

Dialysis reduction in selected patients was performed in our centre prior to the peak incidence of the COVID-19 pandemic in the North West of England, UK. The primary aim was to reduce COVID 19 exposure, transmission between patients and allow for social distancing whilst on the HD unit. It also permitted the formation of a ‘hot’ dialysis unit to cohort all suspected, proven cases and contacts for COVID-19, whereas non-exposed patients could be grouped into ‘cold’ satellite dialysis units. At the onset of this project we began collecting data in a structured way and this paper describes our methodology, outcomes and learning.

## Methods

**Fig. 1:**
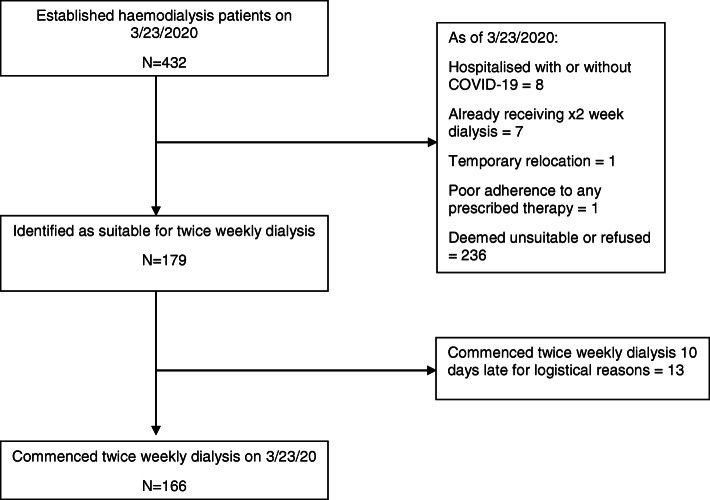
Consort diagram of patient participation

Our regional renal service has a catchment population of 1.55 million people and undertakes in centre haemodialysis for 432 patients in one main centre and 4 outlying satellite centres. The furthest distance between the main centre and satellite centres is 18 miles, which facilitated re-designation of patients without excessive travel times or major inconvenience. In centre HD patients were remotely reviewed (using electronic dialysis care records) by their own nephrologists to determine suitability for twice weekly dialysis (Fig. 1). No definitive inclusion criteria were specified but only named patients dialysis consultants were asked to consider suitability for twice weekly based upon their review of interdialytic weight gain, pre dialysis blood pressure and potassium, residual renal function, comorbidity and functional status. Patient concerns were addressed by the dialysis unit managers and by telephone review with their nephrologist to ensure shared decision making. All patients provided verbal consent to be included within this study. Exclusion criteria included; 1) already receiving twice weekly dialysis; 2) patient refusal 3) highly irregular attendance for dialysis 4) hospitalised on the 23rd March 2020. Patients were transferred to twice weekly dialysis at the beginning of the week commencing 23rd March 2020 and received telephone dietary and fluid advice regarding potential changes in fluid and food intake which may have been necessary. All twice weekly dialysis took place in the satellite centres which were maintained as ‘cold’ sites, whilst the main centre was designated the ‘hot site’ for dialysis of suspected and confirmed COVID-19 patients. The ‘hot’ site also grouped HD patients who were contacts within a COVID-19 household. Patients underwent structured active monitoring of their dialysis parameters between Monday 23rd March 2020 and Monday 20th April 2020. Target weight, pre-dialysis systolic blood pressure (SBP) and pre-dialysis potassium were taken from the first dialysis session of the week preceding conversion for comparative purposes. During week 1 (3/23/20 to 3/29/20) patients were dialysed on Monday and Friday if their usual days were Monday, Wednesday and Friday. Similarly, patients who dialysed on Tuesday, Thursday, Saturday were dialysed on Tuesday and Saturday. During week 2, 3 and 4 patients were dialysed on Monday and Thursday, Tuesday and Friday or Wednesday and Saturday. Dialysis parameters were remotely reviewed at the end of each day for the first dialysis session of each week. Key safety indicators included pre-dialysis SBP, pre-dialysis weight gain, ultrafiltration rate (UFR) and pre-dialysis potassium (K+). Two nephrologists (JT + IE or IA or AH or PE) independently reviewed each patient’s dialysis data and agreed interventions. A pre-dialysis K+ of > 6.5 mmol/L was the only absolute indication to convert back to thrice weekly dialysis. Increased surveillance of dialysis parameters were indicated if any of the following events occurred: SBP > 180 mmHg, UFR > 10 mL/kg/hr., pre-dialysis weight > 5% of target weight and > 3 l ultrafiltration (UF) per session. Dietetic consultation and medical review of both dialysis sessions in that week were undertaken. Pre-dialysis potassium was monitored closely with the following management plan adopted:

**Table:** 

**Pre-dialysis potassium (mmol/l)**	**Action**
**5.7–6.0**	Repeat pre-dialysis potassium at next dialysis session alongside dietetic telephone consultation
**6.1–6.4**	Commence potassium binder (5 g once a day of sodium zirconium cyclosilicate [12]), sodium bicarbonate 1 g 3 times per day [13] and dietetic telephone consultation. Repeat pre-dialysis potassium at next dialysis session. If potassium > 6.0 at next session increase dose of binder by 5 g OD to maximum 15 g OD.

Renal pharmacists and renal dietitians prescribed and managed the prescriptions, patient counselling and hyperkalaemia dietary advice. Dialysis prescriptions (including dialysate potassium) were not changed during this period. All patients were prescribed 4 h of dialysis.

Demographic, comorbidity, biochemical and dialysis adequacy comparisons between patients who continued to receive thrice weekly dialysis and those transferred to twice weekly dialysis were performed. Diagnosis, medication and comorbidity data were taken from coded diagnoses available on hospital and primary care electronic medical records. Usual pre-dialysis blood pressures were calculated using the average of the last readings prior to 3/23/20. Urine output was not contemporaneously measured but patients who self-reported anuria (< 100 mL per day) was recorded. Thirteen patients who commenced twice weekly dialysis after being identified later on during the course of this project were subsequently excluded from analysis in the thrice weekly group at baseline.

Patients who were hospitalised or admitted to the COVID-19 dialysis ‘hot unit’ were transferred to thrice weekly dialysis for the rest of the project. All hospitalisations were recorded after scrutinizing hub hospital (where the main centre is based) admission data and all referrals from surrounding district hospitals for the period 3/23/20 to 4/20/20. Patients were defined as COVID-19 positive after a positive nasal and/or throat COVID-19 PCR. Patients who were transferred back to thrice weekly dialysis completed 3 dialysis sessions in the following 7 days. Cause of death was obtained from death certification and all fatal cases were discussed at a weekly multi-disciplinary mortality and morbidity meeting (including at least 5 nephrologists) to evaluate whether the reduction in dialysis frequency was contributory.

This is an observational report of a strategic and organisational restructuring of dialysis provision in our centre during the COVID 19 pandemic..

### Statistical analysis

Data were analysed using means and standard deviations (parametric data) and medians and interquartile ranges (non-parametric data) where appropriate. Categorical data were compared using chi square test. Continuous data were compared using unpaired T test and Mann U Whitney. Significance of pre-dialysis SBP, potassium and weight changes were compared at each data point (after week1, after week 2 and after week 3) using Mann-Whitney U test. Data were analysed on an as-treated basis. Only patients participating in twice weekly dialysis at each time point were analysed.

## Results

### Baseline characteristics

From a cohort of 432 in-centre dialysis patients 179 (41.4%) were identified as potentially suitable for and agreed to undergo twice weekly dialysis. This manuscript reports on the 166 (38.4%) patients who then commenced twice weekly dialysis on 3/23/20. Patients who were transferred to twice weekly dialysis were more likely to be older, with lower ultrafiltration volumes, greater urea reduction ratio (URR), shorter dialysis vintage and to have lower pre-dialysis phosphate and potassium levels. There was no significant difference in the frequency of primary renal diseases between the two groups and there were significantly less patients with heart failure transferred to twice weekly HD (Table 1).

### Longitudinal changes in dialysis parameters

**Fig. 2:**
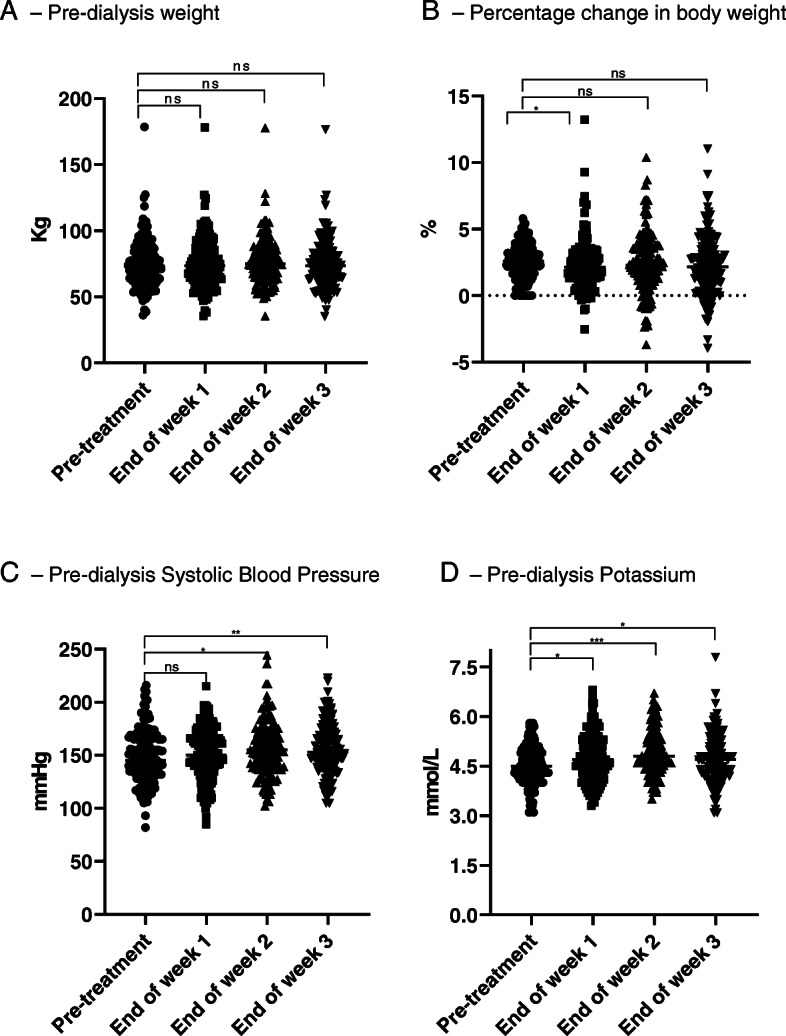
Changes in dialysis parameters in HD patients who remained on twice weekly dialysis. **a** – Pre-dialysis weight. **b** – Percentage change in body weight. **c** – Pre-dialysis Systolic Blood Pressure. **d** – Pre-dialysis Potassium. Statistical significance is shown by Mann-Whitney test: ns = not statistically significant, * *p* < 0.05, **p < 0.01, ***p < 0.001 between twice and thrice weekly HD patients

Dialysis parameters of those patients who remained on a twice weekly dialysis regime demonstrated that pre-dialysis weight and interdialytic percentage increase in body weight remained unchanged (Fig. 2a and b). However, as the weeks progressed there was a significant increase in median SBP and potassium for those who remained on the twice weekly dialysis regime. These findings persisted after excluding patients who were known to be anuric (data not shown). The increase in SBP was apparent after 2 weeks of twice weekly dialysis (Fig. 2c) and the median SBP at the end of 3 weeks of twice weekly dialysis was 153 (140–172) mmHg compared with a median SBP at baseline of 145 (132–165) mmHg. The number of patients with a pre-dialysis SBP > 180 mmHg at baseline and after weeks 1,2 and 3 were 17, 18, 21 and 20, respectively.

There was a significant longitudinal increase in pre-dialysis potassium. This difference was apparent after 1 week of twice weekly dialysis (Fig. 2d). The median potassium at the end of 3 weeks in those patients able to continue twice weekly dialysis was 4.7 mmol/L (4.2–5.2) compared with 4.5 mmol/L (4.1–4.9) at baseline. The number of patients with a pre-dialysis potassium above 6.0 mmol/L was 0, 8, 12 and 6 at baseline and after 1,2 and after 3 weeks of twice weekly dialysis respectively.

### Technique survival

**Fig. 3:**
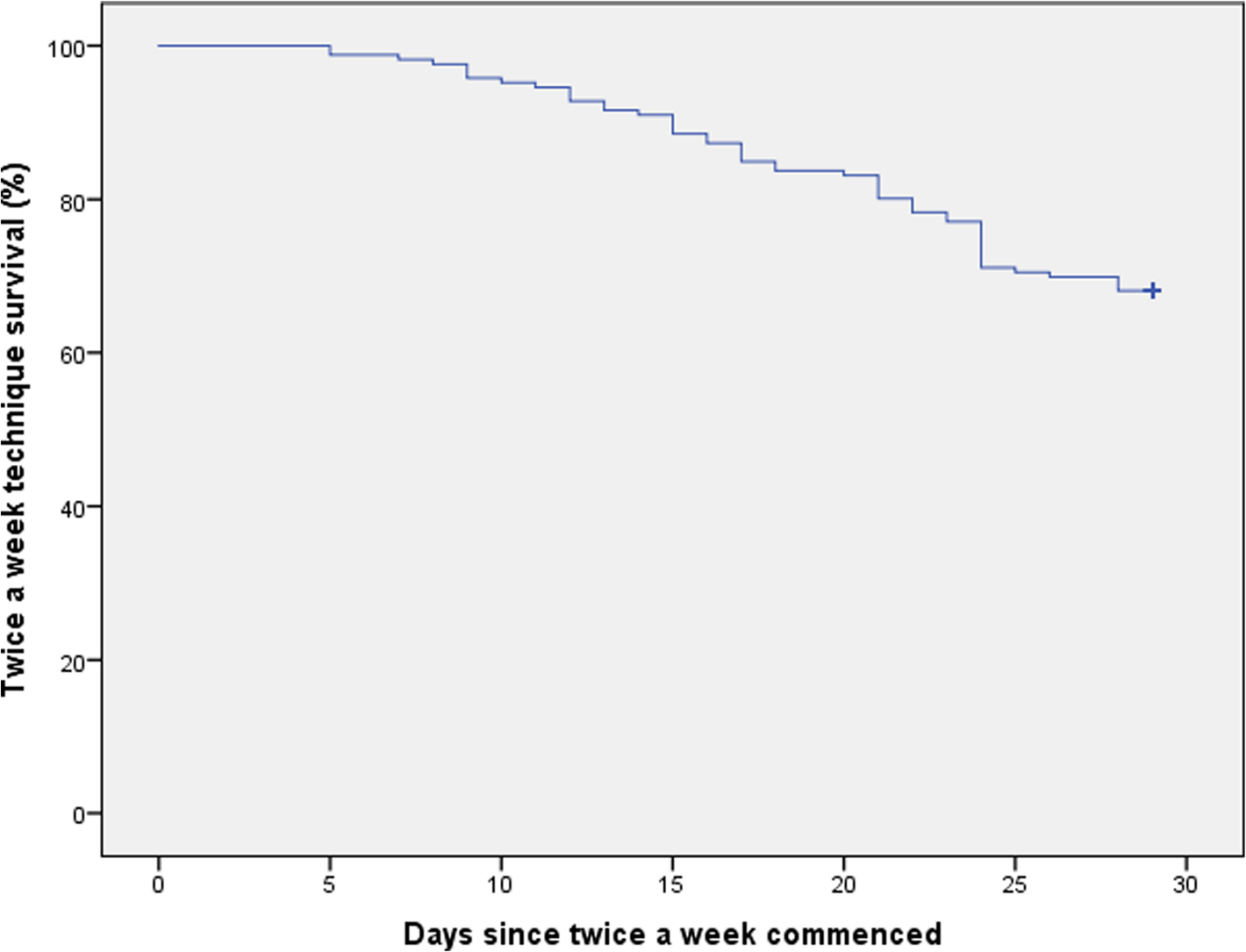
Technique survival curve in eligible population

We determined this by including patients who died (6), patients automatically transferred back to thrice weekly dialysis because of hospitalisation (13) or suspected/confirmed COVID-19 infection (6) as “failing twice weekly dialysis”. There were 113 (68.1%) patients who were able to continue twice weekly dialysis for the whole 4 week period (Fig. 3). This resulted in 452 fewer dialysis sessions potentially minimising 452 potential exposures to COVID-19 both during dialysis and on transport to and from the units. There were 28 patients we electively transferred back to thrice weekly dialysis during the 4 weeks of the project. The indications for transfer back were fluid overload (*n* = 19), hyperkalaemia despite use of binders (*n* = 4), patient’s request (*n* = 4) and compliance concerns (*n* = 1). The characteristics of these 28 patients who we know “failed” twice weekly dialysis due to definite dialysis related reasons (rather than exposure or contraction of COVID-19 for example) are displayed in Table 2.

### Other clinical outcomes

The main outcomes for patients are displayed in Table 3. There were 12 deaths in the entire HD population during these 4 weeks, of whom 6 had been transferred to twice weekly HD. No death was directly related to twice weekly dialysis (Table 4). Two patients died from acute ischaemic events after 12 h but within 48 h of their last dialysis session, 1 patient died from COVID-19 pneumonia, whilst another died from suspected COVID-19 pneumonia. Two patients died at home > 12 h but < 24 h after their last dialysis session; there was no suspicion of COVID-19. One was frail and elderly and the other was an unexpected sudden death that was referred to the coroner. Sudden cardiac death is a common cause of death for patients undergoing dialysis [14]. An association between dialysis reduction to this sudden death was not thought likely based upon dialysis parameters and laboratory results from the preceding dialysis session.

There were proportionately less patients who suffered COVID-19 in the twice weekly group but this difference was not statistically significant. There were no admissions with fluid overload in the twice weekly group during this 4-week project.

**Table 1:** Baseline clinical and laboratorial characteristics of HD patients

	Twice weekly HD (*n* = 166)	Thrice weekly HD (*n* = 236)	*P* value
Age (years)	65.0 [54.8–74.3]	59.0 [49.0–71.0]	**< 0.001**
Sex– Male (%)	107 (64.5)	154 (65.3)	0.870
Ethnicity - White British (%)	110 (66.3)	167 (70.8)	0.339
Pre HD weight (Kg)^a^	73.5 [62.0–85.3]	76.2 [64.2–91.4]	**0.038**
BMI^b^	25.8 [22.9–29.4]	27.5 [23.3–31.6]	0.050
Last URR (%)^c^	71.1 [65.4–75.5]	69.3 [63.1–74.4]	**0.009**
Corrected Calcium^d^ (mmol/L)	2.36 ± 0.18	2.34 ± 0.17	0.283
Phosphate^e^ (mmol/L)	1.56 [1.27–1.89]	1.68 [1.33–2.07]	**0.023**
Parathyroid Hormone^f^ (pmol/L)	26.9 [12.5–56.8]	28.9 [13.2–51.9]	0.361
Haemoglobin^g^ (g/L)	107 ± 16.1	107 ± 17.6	0.873
Potassium^h^ (mmol/L)	4.50 [4.00–5.20]	4.90 [3.20–5.50]	**< 0.001**
Albumin^i^ (g/L)	38.0 [34.1–41.0]	38.5 [35.0–41.0]	0.358
Pre-HD average SBP^j^	148 ± 21.7	147 ± 22.4	0.617
Pre-HD average DBP^k^	74.0 [62.3–84.8]	77.5 [67.0–88.0]	0.082
Post-HD average SBP^l^	139 [126–154]	137 [121–156]	0.980
Post-HD average DBP^m^	72.0 [63.0–80.0]	71.5 [63.8–83.3]	0.584
Average UF^n^ (L)	1.40 [1.00–2.00]	2.00 [1.50–2.70]	**< 0.001**
Anuric Status^o^ (n)	16	N/A	
Dialysis Vintage (months)	21 (6.3–14.5)	29.5 (11.9–261)	**0.013**
Primary Renal Disease			
Diabetic Nephropathy (%)	51 (30.7)	81 (34.3)	0.450
Hypertensive/Renovascular disease (%)	27 (16.3)	30 (12.7)	0.314
Glomerulonephritis (%)	21 (12.7)	31 (13.1)	0.886
ADPKD (%)	15 (9.0)	13 (5.5)	0.172
Urological (%)	13 (7.8)	24 (10.2)	0.425
Pyelonephritis (%)	3 (1.8)	11 (4.7)	0.125
ANCA-associated vasculitis (%)	4 (2.4)	5 (2.1)	0.847
Other (%)	17 (10.2)	16 (6.8)	0.214
Unknown (%)	15 (9.0)	25 (10.6)	0.610
Comorbidity			
Ischaemic heart disease (%)	25 (15.1)	40 (17.0)	0.613
Heart Failure (%)	5 (3.0)	18 (7.6)	**0.049**
CVA (%)	16 (9.7)	35 (14.8)	0.124
Diabetes Mellitus (%)	62 (37.4)	101 (42.8)	0.274
ACEi (%)	30 (18.1)	46 (19.5)	0.721
ARB (%)	19 (11.5)	32 (13.6)	0.532

Results are expressed as mean ± SD, median [IQR; interquartile range] or n (%).*p*-value calculated using unpaired T test for parametric data and Mann-Whitney U Test for non-parametric data. Categorical variables were analysed by Chi-square test. Abbreviations: BMI – Body Mass Index, HD – Haemodialysis, SBP – Systolic Blood Pressure, DBP – Diastolic Blood Pressure, URR – Urea Reduction Ratio, ADPKD – Autosomal Dominant Polycystic Kidney Disease, CVA – Cerebrovascular event, ACEi– Angiotensin converting enzyme inhibitor, ARB - Angiotensin receptor blocker. ^a^missing for 2 patients in twice weekly group. ^b^missing for 2 patients in twice weekly group and 4 patients in thrice weekly group. ^c^missing for 26 patients in twice weekly group and 38 patients in thrice weekly group. ^d^missing for 2 patients in twice weekly group. ^e^missing for 2 patients in twice weekly group. ^f^missing for 4 patients in twice weekly group and 78 patients in thrice weekly group. ^g^missing for 2 patients in twice weekly group. ^h^missing for 2 patients in twice weekly group. ^i^missing for 1 patient in twice weekly group. ^j^Pre-HD average SBP data missing for 4 patients in twice weekly group and 4 patients in 3x week HD group. ^k^missing for 4 patients in twice weekly group and 4 patients in thrice weekly group. ^l^missing for 9 patients in twice weekly group and 10 patients in thrice weekly group. ^m^Post-HD average DBP data missing for 9 patients in twice weekly group and 10 patients in thrice weekly group. ^n^missing for 3 patients in twice weekly group. ^o^missing data 79 patients

**Table 2:** Main clinical outcomes of the project at 4 weeks after treatment change

	Twice weekly HD	Thrice weekly HD	*P* value
URR (%)^a^	72.6 [66.4–77.2]	69.7 [62.7–74.8]	**0.009**
Corrected Calcium^b^ (mmol/L)	2.33 [2.20–2.42]	2.33 [2.22–2.43]	0.921
Phosphate^c^ (mmol/L)	1.77 [1.44]	1.65 [1.33–2.09]	0.117
Potassium^d^ (mmol/L)	4.80 [4.30–5.40]	4.90 [4.40–5.40]	0.329
UF per session (L)	1.50 [1.00–2.03]	2.00 [1.40–2.63]	**< 0.001**
COVID-19 positive (%)	9 (5.4)	20 (8.5)	0.245
Hospitalisations (%)	13 (7.8)	17 (7.2)	0.813
Deaths (%)	6 (3.6)	6 (2.5)	0.535

Results are expressed as median [IQR; interquartile range] or n (%). p-value calculated using Mann-Whitney U Test. Categorical variables were analysed by Chi-square test. Hospitalisation data includes patients who were hospitalised for COVID-19 and prior to death. ^a^missing for 56 patients in twice weekly group and 126 patients in thrice weekly group. ^b^missing for 9 patients in twice weekly group and 46 patients in thrice weekly group. ^c^missing for 9 patients in twice weekly group and 46 patients in thrice weekly group. ^d^missing for 32 patients in thrice weekly group

COVID-19, hospitalisations and deaths % calculated in relation to original number of patients *n* = 166 / *n* = 236. Abbreviations: *URR* Urea reduction ratio, *UF* Ultrafiltration

**Table 3:** Baseline clinical and laboratorial characteristics for twice weekly patients

	Completed full trial (*n* = 113)	Failed twice weekly dialysis (*n* = 28)	*P* value
Age (years)	63.1 ± 14.2	65.1 ± 14.7	0.5257
Sex– Male (%)	76 (67.3)	12 (52.2)	0.1686
Ethnicity - White British (%)	78 (69.0)	17 (73.9)	0.6420
Pre HD weight (Kg)	73.5 [62.0–83.2]	76.4 [71.6–86.6]	0.1545
BMI	25.8 [22.7–28.8]	28.2 [24.5–32.9]	**0.0395**
Last URR (%)^a^	70.6 [66.2–76.7]	72.5 [69.3–74.3]	0.8383
Corrected Calcium (mmol/L)	2.35 ± 0.18	2.36 ± 0.18	0.7784
Phosphate (mmol/L)	1.55 ± 0.44	1.68 ± 0.56	0.2030
Parathyroid Hormone^b^ (pmol/L)	28.3 [12.0–59.2]	25.7 [11.6–36.5]	0.3732
Haemoglobin (g/L)	108 ± 15.5	101 ± 19.9	0.0940
Potassium (mmol/L)	4.52 ± 0.77	4.76 ± 0.84	0.1792
Albumin (g/L)	38.3 ± 4.65	35.0 [32.7–40.0]	0.0557
Pre-HD average SBP	148 ± 21.3	149 ± 21.0	0.8362
Pre-HD average DBP	73.9 ± 15.4	74.8 ± 13.1	0.7889
Post-HD average SBP^c^	138 [125–155]	134 [125–150]	0.5987
Post-HD average DBP^d^	71.0 [62.5–80.0]	72.0 [64.0–79.0]	0.8243
Average UF (L)	1.30 [1.00–2.00]	2.20 [1.40–2.60]	**0.0007**
Dialysis Vintage (months)	19.2 (5.3–45.2)	20.7 (8.0–61.4)	0.5270
Primary Renal Disease			
Diabetic Nephropathy (%)	35 (31.0)	9 (39.1)	0.4508
Hypertensive/Renovascular disease (%)	20 (17.7)	4 (17.4)	0.9727
Glomerulonephritis (%)	13 (11.5)	5 (21.7)	0.1897
ADPKD (%)	11 (9.7)	2 (8.7)	0.8821
Urological (%)	9 (8.0)	1 (4.3)	0.5375
Pyelonephritis (%)	2 (1.8)	0 (0.0)	0.5183
ANCA-associated vasculitis (%)	2 (1.8)	0 (0.0)	0.5183
Other (%)	11 (9.7)	0 (0.0)	0.1207
Unknown (%)	10 (8.8)	2 (8.7)	0.9877
Comorbidity			
Ischaemic heart disease (%)	21 (18.6)	2 (8.7)	0.2502
Heart Failure (%)	4 (3.5)	1 (4.3)	0.8523
CVA (%)	8 (7.1)	6 (26.1)	**0.0065**
Diabetes Mellitus (%)	48 (42.5)	10 (43.5)	0.9298
ACEi (%)	24 (21.2)	5 (21.7)	0.9576
ARB (%)	18 (15.9)	0 (0.0)	**0.0408**

Results are expressed as mean ± SD, median [IQR; interquartile range] or n (%).p-value calculated using unpaired T test for parametric data and Mann-Whitney U Test for non-parametric data. Categorical variables were analysed by Chi-square test. Abbreviations: BMI – Body Mass Index, HD – Haemodialysis, SBP – Systolic Blood Pressure, DBP – Diastolic Blood Pressure, URR – Urea Reduction Ratio, ADPKD – Autosomal Dominant Polycystic Kidney Disease, CVA – Cerebrovascular event, ACEi– Angiotensin converting enzyme inhibitor, ARB - Angiotensin receptor blocker. ^a^missing for 13 patients in completed full twice weekly group and 5 patients in failed twice weekly group. ^b^missing for 1 patient in failed twice weekly group. ^c^missing for 4 patients in completed full twice weekly group. ^d^missing for 4 patients in completed full twice weekly group

**Table 4:** Causes of death

	Twice weekly HD	Thrice weekly HD
COVID Pneumonia	1	4
Cardiovascular Disease	2	0
Myeloma	0	1
Natural Causes	0	1
Chronic Kidney Disease/End Stage Renal Disease	2	0
Uncertain (sudden death at home)^a^	1	0
Total	6	6

Causes of death were taken from death certification records. All deaths were discussed in mortality and morbidity meetings to ensure there was no direct relationship between dialysis reduction and cause of death. ^a^This patient has been referred for a coroner’s investigation (unrelated to twice weekly dialysis)

### Hyperkalaemia interventions

There were 19 patients who received a prescription of sodium bicarbonate 1 g thrice daily and sodium zirconium cyclosilicate alongside further dietetic advice. Two of these patients died (1 with normal potassium on admission and 1 with normal pre-dialysis potassium on the previous dialysis session (4.8 mmol/L). 7/19 patients were subsequently transferred back to thrice weekly dialysis and 1 patient did not tolerate either medication. Nine patients continued to take the medications and dialyse twice weekly. Hyperkalaemia interventions reduced pre-dialysis potassium by 0.9 mmol/L (IQR 0.8–1.4); one patient’s potassium increased following intervention.

## Discussion

This analysis found that the majority of patients who are deemed suitable to temporarily convert to twice weekly dialysis were able to safely dialyse twice weekly for at least 1 month during the COVID-19 pandemic, enabling safer grouping of patients to reduce potential viral exposure and transmission and ease service demands which may have been exacerbated by staff sickness. However, this was only possible with very close monitoring via dedicated clinician time and through the use of digital technology allowing remote monitoring of biochemistry and dialysis parameters. The necessity for close monitoring can be demonstrated for two reasons. Firstly, longitudinal assessment of dialysis parameters demonstrated statistically significant increases in pre-dialysis systolic blood pressures and pre-dialysis potassium in those patients who continued to receive twice weekly dialysis, although overall these parameters remained well within ‘safe’ limits. Secondly, it was also noted that the rate of patient transfer back to thrice weekly dialysis was constant throughout the project at around 3–4% per week.

Rising SBP despite no significant increase in pre-dialysis weight suggests that blood pressure changes were not necessarily related to increases in extracellular blood volume (ECV). This finding is not surprising given that more frequent dialysis has been shown to improve blood pressure control through various mechanisms. These include reduced ECV, increased sodium removal, reduced sympathetic tone and removal of vasoactive factors which may be driving hypertension [15–17]. There were similar numbers of patients with a pre-dialysis SBP > 180 mmHg before the project commenced compared with at the end of this twice weekly dialysis project (17 versus 20). A value of SBP > 180 mmHg as a trigger for closer dialysis parameter observation and possible conversion back to thrice weekly after the next dialysis session was based upon evidence that this value delineates an increased mortality risk in dialysis patients, although this evidence is conflicting [18].

There was little difference in the UF volumes in the twice weekly patients at baseline compared with those still maintaining on the twice weekly protocol at the end of the 4-week period (1.4 (1.0–2.0) litres per session compared with 1.5 (1.0–2.0) litres per session). However, this excludes the 19 (13.5%) patients who were transferred back to thrice weekly because of fluid-related issues. Table 3 demonstrates that patients with higher ultrafiltration at baseline were those who could not manage twice weekly dialysis for a 4 week period.

Due to reduced weekly dialysis time it is unsurprising that the median pre-dialysis potassium significantly increased every week. A pre dialysis potassium > 6.0 mmol/L has been suggested as a threshold whereby mortality risk substantially increases [19]. However only 6 patients had a pre-dialysis potassium above 6.0 mmol/L and 75% of the patients had a pre-dialysis potassium < 5.4 mmol/L at the end of this project. This was the same as in the thrice weekly population. Only 2 of the 6 patients with this degree of hyperkalaemia had previously had a pre-dialysis potassium above 6.0 mmol/L during the entire project. We had made no changes to dialysate potassium concentrations because recent evidence has suggested a higher mortality risk when patients are dialysed against a low potassium dialysate (1mEql/l), particularly those patients with a higher serum potassium [20]. The use of potassium binders, sodium bicarbonate and responsive dietetic consultations mitigated against the need to increase dialysis session frequency in 9 patients. The role of potassium binders to reduce hyperkalaemia events and major adverse cardiovascular events in dialysis patients has not been researched in any randomised control study [12]. Potassium profiling was not used in this study but could be an option to mitigate hyperkalaemia in centres where this is an option [21].

There were no significant differences between hospitalisations, COVID-19 infections and deaths between the two groups although the twice weekly group were on average 6 years older than the thrice weekly group, perhaps representing a more at-risk group, although they had lower prevalence of heart failure. There was no definite evidence that any of the 6 deaths in the twice weekly dialysis group were caused by a reduction in dialysis frequency. There were no hospitalisations for fluid overload in the twice weekly group.

The analysis of this 4-week period of change in dialysis protocol is not intended to re-energise the debate over long-term dialysis frequency and dialysis dose but provides a potential methodology to appropriately and safely rationalise dialysis resources during a health service crisis such as the current pandemic. Although we are not recommending generalisation of our approach as health care management differs markedly throughout the world, it may have implications for other countries where dialysis resources are limited. Although we did not have definitive inclusion criteria, we have retrospectively surveyed the clinicians involved and together with the findings of this study we would suggest that short term switch to twice weekly dialysis is most appropriate for;

**Table:** 

● Elderly patients ● Patients with lower ultrafiltration requirements/ higher residual renal function ● Patients whose pre-dialysis phosphate levels are within the normal range ● Patients whose pre-dialysis potassium levels are well within normal range ● Patients who share the decision to switch to twice weekly dialysis	

We investigated complete conversion of patients to twice weekly dialysis. Future studies could investigate the impact of alternating twice to thrice weekly dialysis in selected groups of patients to minimise COVID-19 exposure but maintain satisfactory dialysis parameters. At this stage, having passed the first peak of the COVID-19 pandemic in our geographical region, all our in-centre haemodialysis patients are now being individually reassessed for their suitability and preference for either home haemodialysis or peritoneal dialysis, the latter with particular relevance to patients with residual urine output currently treated with twice weekly haemodialysis. Patients who will not be suitable or do not wish to be transferred to home therapies and do not have significant formally quantified residual renal function will return to thrice weekly dialysis in a planned fashion over the next few weeks [22].

## Conclusions

In conclusion, the COVID-19 pandemic is an international exceptional health crisis. This project demonstrates that reorganisation of dialysis provision for selected patients has the potential to protect patients and clinical staff. Patient selection and careful real-time monitoring can ensure that abrupt changes in dialysis provision are safe.

### Limitations

This analysis did not fully evaluate dialysis adequacy which is reported to influence long term outcomes of HD as this project was primarily designed to overcome the short-term challenge of dialysis provision during a pandemic. It was not possible to perform contemporaneous measurements of residual renal function due to the short lead time of project set-up and there was considerable missing data regarding anuric status (47.6%). Regular measurement of residual renal function within the whole dialysis cohort would undoubtedly aid in decision making to determine suitability to convert to twice weekly haemodialysis at short notice. There was no measure of concordance with interventions (i.e. compliance with potassium binder medication) for hyperkalaemia although all but one patient’s potassium fell after its initiation. Whilst patient’s views were continually monitored during this period there were no quality of life or intradialytic health related quality of life symptoms recorded throughout the 4-week period.

## Data Availability

All data generated or analysed during this study are included in this published article.

## Abbreviations

ACEi: Angiotensin Converting Enzyme inhibitor
ADPKD: Autosomal Dominant Polycystic Kidney Disease
ARB: Angiotensin receptor blocker
BMI: Body Mass Index
COVID-19: Coronavirus disease
CVA: Cerebrovascular event
HD: Haemodialysis
DBP: Diastolic Blood Pressure
ECV: Extracellular blood volume
IQR: Interquartile Range
K^+^: Potassium
OD: Once Daily
SBP: Systolic Blood Pressure
UF: Ultrafiltration
UFR: Ultrafiltration Rate
URR: Urea Reduction Ratio
